# Non-Destructive Detection of Pentachlorophenol Residues in Historical Wooden Objects

**DOI:** 10.3390/polym13071052

**Published:** 2021-03-27

**Authors:** Ida Kraševec, Nataša Nemeček, Maja Lozar Štamcar, Irena Kralj Cigić, Helena Prosen

**Affiliations:** 1 Faculty of Chemistry and Chemical Technology, University of Ljubljana, 1000 Ljubljana, Slovenia; ida.krasevec@fkkt.uni-lj.si (I.K.); helena.prosen@fkkt.uni-lj.si (H.P.); 2 National Museum of Slovenia, 1000 Ljubljana, Slovenia; natasa.nemecek@nms.si (N.N.); maja.lozar@nms.si (M.L.Š.)

**Keywords:** pentachlorophenol, wood, museum collections, solid-phase microextraction, headspace, contact SPME, GC–MS, non-destructive method

## Abstract

Wood is a natural polymeric material that is an important constituent of many heritage collections. Because of its susceptibility to biodegradation, it is often chemically treated with substances that can be harmful to human health. One of the most widely used wood preservatives was pentachlorophenol (PCP), which is still present in museum objects today, although its use has been restricted for about forty years. The development of non-destructive methods for its determination, suitable for the analysis of valuable objects, is therefore of great importance. In this work, two non-destructive solid-phase microextraction (SPME) methods were developed and optimized, using either headspace or contact mode. They were compared with a destructive solvent extraction method and found to be suitable for quantification in the range of 7.5 to 75 mg PCP/kg wood at room temperature. The developed semi-quantitative methods were applied in the wooden furniture depot of National Museum of Slovenia. PCP was detected inside two furniture objects using headspace mode. The pesticide lindane was also detected in one object. The indoor air of the depot with furniture was also sampled with HS SPME, and traces of PCP were found. According to the results, SPME methods are suitable for the detection of PCP residues in museum objects and in the environment.

## 1. Introduction

Wood is a natural polymeric material, composed of cellulose (40–50%), hemicellulose (15–35%), lignin (20–35%), and small amounts of other constituents [1]. Due to its versatility and workability, it has been used by humanity since the beginnings of culture, to make tools, weapons, decorations, sacred objects, buildings, furniture, and countless other products. It is therefore an important part of historical and art collections around the world that institutions try to retain for our descendants. Wood is sensitive to degradation not only due to environmental causes (temperature, humidity, light, and fire) but also due to biological factors (insects and fungi) [2,3,4,5]. To reduce the effects of degradation as much as possible, wooden objects should ideally be stored in a dark, anaerobic location, at 4.5 °C and 50% relative humidity (RH) [5], which is not always achievable in practice. Therefore, most institutions focus only on the most damaging of these parameters—humidity, since its fluctuations strongly influence the swelling and shrinking of wood (causing damage at fluctuations over 10% RH), while its high average value (above 70%) promotes the growth of molds and fungi [5].

The risk of wood biodegradation can be further reduced by chemical treatment, applied either preventively or after infestation already occurred. Various chemicals have been used for this purpose. As early as 1920, the use of paraffin oil, turpentine, petrol ether, HgCl_2_, phenol, and creosote or fumigation with HCN, CS_2_, SO_2_, and CCl_4_ are stated as treatments for insect infestation [6]. Later in the 20th century, a wide range of organic pesticides have been used as wood preservatives, including pentachlorophenol (PCP), DDT, lindane, dieldrin, aldrin, organophosphates, carbamates, pyrethroids (cypermethrin), methylbromide, quaternary ammonium compounds, and naphthalene [1,3,7,8,9,10]. Among the inorganics, compounds such as chromated copper arsenate (CCA), copper–chrome–borate, borates (boric acid, borax), and sulfuryl fluoride were used [1,3,7,11]. Many of these compounds were later proven harmful to the environment or human health, and their use was reduced and restricted [10,11,12]. With the EU directive BPD 98/8/EC, the number of permitted substances for wood protection was reduced to 81 in 2006 [13]. However, many of these compounds can persist in the museum environment, as the stored objects have been treated according to the—at the time—latest practices throughout their lifetime, but have not been decontaminated since.

Pentachlorophenol (PCP) is an organochlorine pesticide that was developed in the 1930s specifically for wood protection and was in widespread use until the 1980s [14,15]. Unlike some other pesticides (e.g., DDT), PCP does not cause visual changes of treated objects (crystal growth, color change) [12]; therefore, it was considered a good choice by many conservators and was often applied as a protection to wooden objects in museums in the form of a sprayed solution or by immersion. PCP is a volatile (vapor pressure at 20 °C is 1.1 × 10^−4^ mmHg [16]), acidic (p*K*_a_ = 4.8 [17]), water soluble compound that can also adsorb to soil and sediments [15,16]. The phenolic group responsible for the acidic properties also leads to chemical bonding to the functional groups of wood lignin, not only on the surface, but throughout the wood structure [17]. In addition to wood preservation, it has also been commonly used as a fungicide, herbicide, and insecticide in the leather and paper industry, and agriculture [15]. Due to its acute and chronic toxicity (with harmful effects on liver, kidneys, lungs, and nervous and immune systems) and probable carcinogenicity to humans, its production and use today is prohibited or heavily restricted (e.g., for utility poles, railroad ties) worldwide [15,18,19], including in Slovenia [20]. It has been detected in various environmental and biological matrices: air [19,21], dust [19,22,23], water [24,25,26], soil [27], sediments [28], animals [18,29], plants [30], and human fluids [28,31,32,33], although concentrations in both the environment and the human tissues have been found to be decreasing over the recent decades [34].

In museum collections in general, about 90 different pesticides are known to have been used [35], but their use is often not well documented, and the risk associated with handling the objects by the staff is poorly understood. In addition to direct skin contact, exposure can also occur through inhalation of dust particles or volatilized compounds, to which museum visitors are also vulnerable, albeit to a lesser extent [12,36]. Therefore, the investigation of the pesticides persisting in museum objects is important from the health perspective.

For any analysis of museum objects, non-invasive (not requiring a removal of a solid sample, leaving the object in the same state as before sampling) and non-destructive sampling techniques are preferred to keep the integrity of the object. When absolutely unavoidable, only imperceptibly small samples can be removed (i.e., micro-destructive techniques), but such samples may not be representative of the object as a whole [37]. Therefore, in addition to various in-situ spectrometric techniques (UV-VIS, IR, Raman, XRF) [3,12,38], sampling of air (on polyurethane foam or Tenax tubes [9,22,23,39]) and dust, as well as the occasional surface swab, are considered acceptable in most collections. In recent years, solid-phase microextraction (SPME) sampling is often used for various investigations in the field of heritage science [40,41,42,43].

In various wood samples, PCP has been most commonly determined by solvent extraction and chromatographic analysis [17,38,44,45,46,47,48,49,50], resulting in low detection limits due to preconcentration. Such methods are destructive, but have been used on wooden art objects in Belgium [51] and Germany [22], where small fragments were removed for analysis. In one case, non-destructive headspace SPME sampling of wood pieces has been performed [52] after their removal into sampling containers.

In this work, our aim was to develop a SPME method for sampling of PCP on large historical wood objects, as it is a simple, non-destructive, in-situ method. Two different modes of sampling were tested and compared: headspace (HS) and contact mode. In addition, the developed method was compared on model wood samples with a destructive approach: solvent extraction followed by an optional solid-phase extraction clean-up step and either HPLC-UV or GC–MS analysis. Finally, the method was used to estimate PCP concentrations in museum furniture storage and to assess the risk to the museum employees.

## 2. Materials and Methods

### 2.1. Model Wood Samples Preparation

Untreated, dry beech and spruce wood was cut into small pieces with an average mass of 1.5 g. PCP (>98%, Aldrich, St. Louis, MO, USA) was dissolved in acetone (HPLC grade, Sigma-Aldrich, St. Louis, MO, USA) and pipetted onto the largest surfaces of the wood pieces, thereby imitating the application by spraying. The samples were left in the fume hood for 24 h to allow the solvent to evaporate completely. The prepared PCP amounts in the wood ranged from 7.5 to 75 mg/kg.

### 2.2. SPME Methods

For all SPME samplings, Supelco (Bellefonte, PA, USA) SPME fibers with 100 μm PDMS coating were used, pre-conditioned for 30 min at 250 °C. For headspace sampling, the model samples were enclosed into 20 mL headspace vials for 24 h at room temperature (24 ± 1 °C) and then sampled with SPME at the same temperature for 40 min. For absolute calibration, 5 mL of PCP water solutions were used as the sample.

In the contact SPME mode, the fiber was placed onto a horizontal wood sample in such a way that maximum surface contact between the fiber and the sample was possible. The sampling proceeded for 30 min at room temperature on the same surface of the model wood samples, to which the PCP solution was previously applied.

After both sampling modes, the fibers were desorbed at 250 °C in the GC–MS injector.

### 2.3. Solvent Extraction and SPE Method

For extraction, the model samples were completely immersed into the solvent for 24 h in closed glass vessels. Acetone extracts were concentrated to exactly 5 mL using a vacuum concentrator (RVC 2-18, Christ, Osterode am Harz, Germany) at 40 °C. and analyzed by GC–MS or diluted 1:1 with ultrapure water (MQ, MilliQ Water System, Millipore, Burlington, MA, USA) and analyzed by HPLC-UV.

Beech model samples were also extracted with a 1:1 mixture of acetone and 1 M H_2_SO_4_ for 24 h. The extract was neutralized with NaOH, and the acetone was removed with the vacuum concentrator. It was diluted with MQ and further cleaned with the following SPE procedure: LC-18 cartridges (Supelco, Bellefonte, PA, USA; 1 g of sorbent) were conditioned with 6 mL of methanol (HPLC grade, Sigma-Aldrich, St. Louis, MO, USA) and MQ in succession, then the diluted extract was applied. The cartridge was rinsed with 5 mL of MQ and eluted with 5 mL of methanol. Finally, the extract was dried to the final volume of 1 mL, which was analyzed by HPLC-UV.

### 2.4. HPLC-UV Analysis

An Agilent Technologies (Santa Clara, CA, USA) HPLC instrument 1100 Series with a quaternary pump and a diode-array detector was used. A Kromasil Eternity-5-C18 (100 mm × 4.6 mm, 5 μm; Sigma-Aldrich, St. Louis, MO, USA) column was used, injection volume was 5 μL, and detection wavelength 254 nm. The gradient elution program was as follows: the initial ratio of 30/70 acetonitrile (HPLC grade, Sigma-Aldrich, St. Louis, MO, USA) and 0.01 M H_3_PO_4_ (85% *p.a.*, Merck, Darmstadt, Germany) in MQ linearly increased to 80/20 acetonitrile and 0.01 M H_3_PO_4_ in 12 min, then remained constant for 3 min.

### 2.5. GC–MS Analysis

A Thermo Scientific (Waltham, MA, USA) GC–MS instrument was used, consisting of a Focus GC gas chromatograph and an ISQ quadrupole mass spectrometer. The injector at 250 °C was in splitless mode; either 1 μL of solution was injected or a SPME fiber was inserted for 10 min. A DB-5MS column (30 m × 0.25 mm, 0.25 μm; Agilent J&W, Santa Clara, CA, USA) and helium as the carrier gas with the flow rate of 0.8 mL/min were used. The temperature program started at 50 °C (held for 2 min), increased to 150 °C at a rate of 20 °C/min, then increased to 300 °C at a rate of 30 °C/min, and remained constant for 5 min. Electron ionization (70 eV) was used in MS and scanning in the *m/z* range 30–550 amu in total ion current mode (TIC), or alternatively, *m/z* 167, 264, 266, and 268 amu were chosen in selected ion monitoring (SIM) mode. A solvent delay of 4 min was applied. MS transfer line was heated to 300 °C, and all compounds were identified using Mass Spectral Library NIST05.

### 2.6. Sampling in the Museum Depot

The developed methods were tested in the depot of historical furniture at the National Museum of Slovenia, where the constant indoor air conditions are 18 °C and 40% relative humidity. Five objects of interest (OBJ1-5) were selected by the conservators and curators (further described in Section 3.4.) and investigated with HS SPME, contact SPME, or surface swabbing with acetone on cotton wool. A small piece of OBJ2 was sacrificed for acetone extraction after headspace sampling in a vial. The air in the depot was sampled at three different locations, where the SPME fibers were exposed for 4 h.

## 3. Results and Discussion

### 3.1. Model Wood Samples

**Figure 1:**
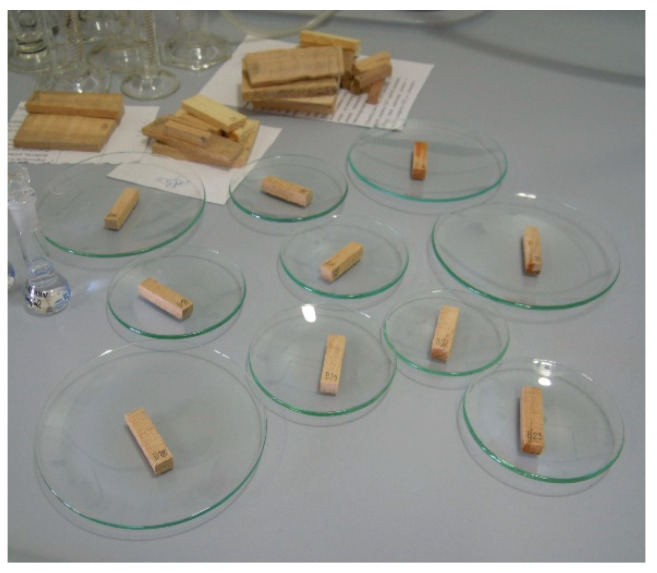
Application of PCP in acetone to model wood samples.

PCP was applied to model wood samples in acetone solution, with the amount of solvent optimized to completely cover the model samples (Figure 1). According to the literature [12,53], such application results in higher pesticide concentrations at the surface due to a lower penetration depth, which would make the analyte accessible for analysis with SPME. This type of application was chosen because it would reproduce the common PCP application to large objects by spraying. Application by full immersion would be unlikely for furniture objects, so this procedure was not used for model samples.

The amount of PCP on the model wood samples decreased with time since sample preparation. The main reason was probably evaporation of PCP from the wood, possibly also redistribution into the inner wood structure. No degradation products were observed during chromatographic analyses. The effect of evaporation was reduced by maintaining a time interval of 24 h between PCP application and sampling.

### 3.2. Solvent Extraction

Two extraction solvents were tested on model wood samples: pure acetone and a 1:1 mixture of acetone and 1 M H_2_SO_4_ (the solvents were selected based on literature [17,48]). The procedure with acid included an additional SPE clean-up step that was optimized separately using aqueous solutions with low concentrations (0.1–0.4 mg/L) of PCP (resulting in average SPE recoveries of (112 ± 12)%). For model wood samples with a PCP concentration of 90 mg/kg, the average extraction recovery was (84 ± 11)% with acetone and (66 ± 5)% with the mixture of acetone and H_2_SO_4_ (1:1) combined with SPE. The extraction recoveries were comparable to those reported in the literature, where PCP recoveries from wood with different solvent systems ranged 60–100% [44], 75–90% [38], 82–98% [48], or above 98% [17].

The lower extraction recovery with acetone–acid extraction was an unexpected result, since other researchers have shown that the addition of sulfuric acid improves PCP extraction from wood due to the additional extraction of PCP in the form of a sodium salt [17]. In our case, the lower recovery with the acetone–acid mixture could be due to an increased number of processing steps or due to an intensive drying step. The presence of the acid increased the repeatability of the extraction; %RSD for at least three replicates was 11% for acetone extraction and 5% for acetone–acid extraction.

**Figure 2:**
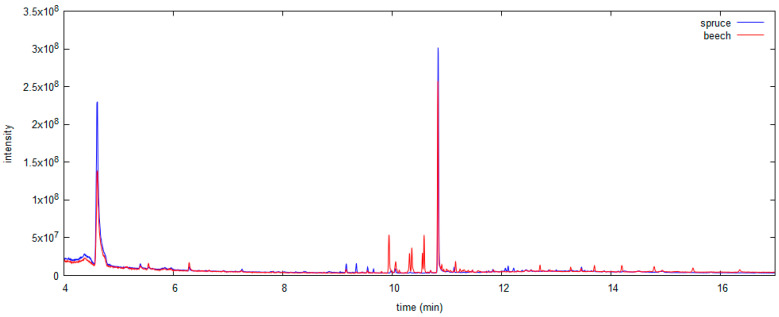
GC–MS TIC chromatograms of acetone extracts from beech and spruce model wood samples (PCP at 10.8 min).

GC–MS chromatograms of acetone extracts from beech and spruce model wood samples treated with PCP are shown in Figure 2. In addition to the peak for PCP (10.8 min), other peaks were observed that presumably represent compounds naturally presented in different wood types. Additionally, comparison of the HPLC-UV chromatograms (at 254 nm) of the extracts of model wood samples revealed some additional peaks of unidentified compounds in case of extraction with acid; therefore, this solvent was considered less selective.

**Figure 3:**
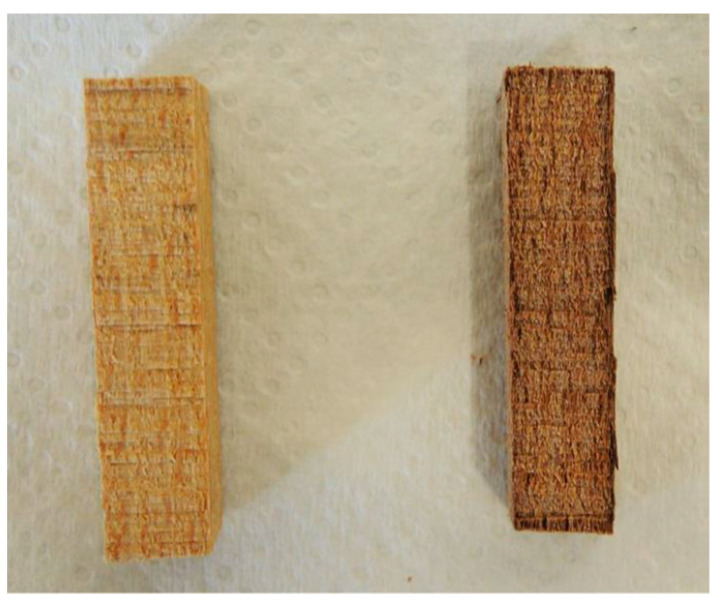
Color change of model samples after solvent extraction: left—acetone, right—acetone with sulfuric acid.

The solvent extractions caused visible damage to the model wood samples in the form of color change. The effect was particularly intense in the samples extracted with sulfuric acid: their surface darkened and acquired a reddish hue (Figure 3). This underlines the main problem of observable damage to non-sacrificial samples caused by the use of solvent extraction methods. The damage can be even more pronounced when paints or varnishes are applied to the wood surface, as these materials are usually sensitive to the solvents.

### 3.3. SPME Sampling

#### 3.3.1. SPME in Headspace Mode

Headspace (HS) SPME sampling at elevated temperature was investigated on model wood samples and resulted in high signals for PCP at both 40 and 50 °C even at a short sampling time of 10 min. Therefore, all further HS samplings were done at room temperature 24 ± 1 °C, as a sufficiently intense signal for PCP was observed, and these conditions are comparable to a museum environment.

The sampling time at room temperature was optimized using the prepared model wood samples with a PCP content of 7.5 mg/kg. Times between 10 and 50 min were tested, with the analyte signals increasing with longer sampling time. The analyte distribution between the headspace and the fiber coating was in the equilibrium approximately at 50 min. A sampling time of 40 min was selected, because no significant increase in the analyte signal occurred with longer sampling time.

At the selected conditions, the linearity of the signal in HS sampling was investigated. Headspaces of model wood samples with PCP concentration between 7.5 and 75 mg/kg were sampled in duplicate. The R^2^ of the calibration curve was 0.9524, which was considered acceptable due to the manual sampling and the two equilibria involved (Table 1).

To estimate the concentration of PCP in the air above the model wood samples, HS SPME sampling of water solutions with PCP concentrations of 10–75 mg/L was performed. According to Henry’s law for dilute solutions, the ratio between the amount of a species in the gas phase and in the aqueous phase is constant at constant temperature. The Henry’s law volatility constant, K_Hpc_ varies for PCP in different literature sources. The value of 2.44 × 10^−8^ (atm·m^3^)/mol for PCP in water at 22 °C according to [54] was selected. Following the equations by Sander [55], a dimensionless constant H_cc_ = 9.99 × 10^5^ at 24 °C was calculated, and further, also the PCP concentrations in air above the water solutions, using the equation γ_g_ = γ_aq_/H_cc_. The correlation between the calculated PCP concentration in the air (c) and the GC–MS response after HS SPME sampling was linear in the range of 0.01–0.75 mg/m^3^, with the correlation equation Ar = 1.28 × 10^10^ c − 7.15 × 10^6^ and the correlation coefficient R^2^ = 0.994.

Through this calibration, it was estimated that a model wood sample with a PCP content of 50 mg/kg would result in a PCP concentration of 4 ng/m^3^ in air in a closed vial used for HS sampling, assuming that the PCP distribution between wood and air is similar to the distribution between water and air and that the SPME response remains linear even at lower air concentrations.

#### 3.3.2. SPME in Contact Mode

**Figure 4:**
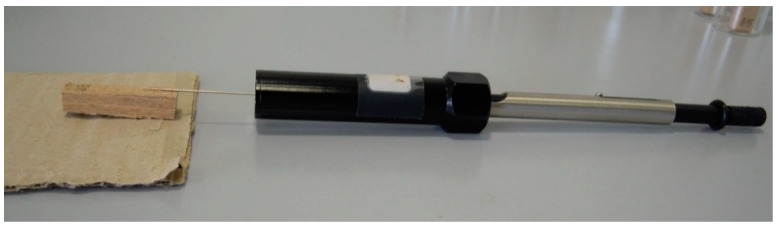
SPME sampling of model wood sample in contact mode.

In order to focus the surface area from which the analyte is collected, the SPME fiber was placed into direct contact with the model wood samples (Figure 4). Such sampling would be useful in cases where the investigated objects are large and cannot be placed in an enclosed environment.

**Figure 5:**
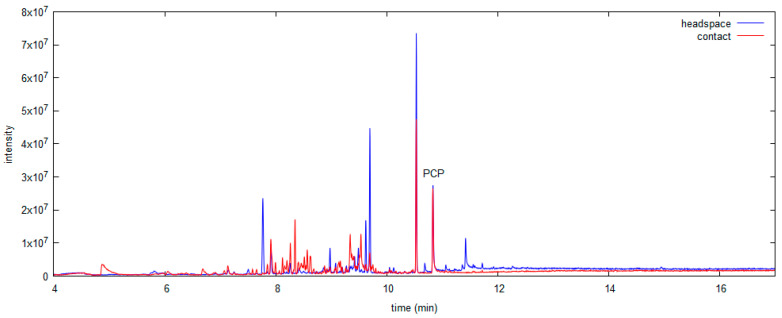
Comparison of GC–MS chromatograms (TIC) of a model wood sample in HS and contact SPME mode.

At room temperature, comparable analyte signals to those by HS sampling were obtained with contact SPME after 30 min; therefore, this sampling time was used in all further experiments. The R^2^ of the calibration curve in the same PCP concentration range in model wood samples was 0.8993, slightly lower than that of HS SPME (Table 1). The linearity of this calibration is significantly lower than for HS sampling, mainly due to a higher %RSD of the complete method (Table 1). The amount of analyte collected by contact SPME depends on its distribution on the surface, which is not entirely homogeneous. It was also noticed in our study, that the PCP amount determined during contact SPME sampling from the front surface (where the PCP solution was applied to prepare the model samples) exceeded the amount determined by sampling from the back surface by approximately 10%. Because the analyte also penetrated deeper into the wood structure, the amount of PCP determined with contact SPME on the surface likely underestimated the concentration of PCP in the entire sample. An important parameter that also caused the higher %RSD of this method was the air movement above the fiber, which was not controlled. Compared to the closed vial for HS sampling, air velocities were higher during contact sampling, which may affect the distribution of the analyte onto the fiber. In contact mode, more compounds were extracted than in HS mode; therefore, HS can be considered more selective (Figure 5). HS sampling has better repeatability than contact sampling, which was expected. Due to this and the fact that the linear fit was lower in contact mode, the limit of detection was lower in HS (20 mg PCP/kg wood) than in contact mode (30 mg PCP/kg wood).

For both SPME methods, no observable damage was caused to the samples (in contrast to the solvent extractions); therefore, these methods can be considered non-destructive. Some compounds appear in the chromatograms after both sampling modes, but with different signal intensities (as shown in Figure 5). Comparison with chromatograms after solvent extraction (Figure 2) shows that different compounds were extracted by the two methods, and the retention times of the majority of peaks suggest that a larger amount of low volatility compounds was extracted with acetone than with SPME, which is predictable. The highest intensity of PCP signal (at the same concentration in wood sample) was observed in the chromatogram of an acetone extract; therefore, the solvent method would be considered to have lower LOD than both SPME methods. From the instrumental limit of detection and the extraction recovery, the LOD for the acetone extraction method was estimated to be 10 mg PCP/kg wood, which is only slightly better than for SPME methods (Table 1). Repeatability was comparable between all the developed methods and ranged 5–16% (Table 1).

Several methods for the non-destructive analysis of PCP in wood have been described in literature: NIR, IR, and Raman spectroscopy in combination with PCA analysis have been shown to be capable of discriminating between organic pollutants in waste wood, but their quantification was not possible [3,56]. XRF cannot identify individual organochlorine compounds, but their distribution and some quantitative data can be obtained by detecting chlorine [12]. In a study of wood fragments using a SPME–GC–FAIMS method, PCP was identified, but again quantification was not possible [52]. In comparison to these methods, the SPME methods developed in this work are suitable for accurate identification of PCP and can also provide limited quantitative information, while meeting the requirements of non-destructive and non-invasive analysis.

### 3.4. Application of the Sampling Methods to Museum Objects

Five wooden objects were selected from the National Museum of Slovenia furniture collection, which includes a large number of objects dated from 15th to 20th century. According to the conservation documentation, chlorinated pesticides, including PCP and lindane, were used in the collection until 1990s. After that, permethrin, carbamates, and borates were used, while in recent years, the main method of protection is anoxia.

The objects and the sampling methods used are described in Table 2. In addition, a swab with acetone on cotton wool was collected in non-lacquered, inconspicuous locations on all objects as an alternative to solvent extraction and for comparison with SPME.

**Table 2:** Description of the museum objects examined, sampling methods used and the PCP detection.

	Object Description	Sampling	PCP Detection
**OBJ1**	Small cabinet with doors and a drawer, open top surface.	An already detached trim collected (*m* = 2.709 g); contact SPME on both largest surfaces.	<LOD
**OBJ2**	Nightstand with tall narrow legs.	An already detached piece of wood collected (*m* = 0.201 g); HS SPME in vial and acetone extraction.	Not detected
**OBJ3**	Black chest of drawers, with nine drawers (Figure 6).	HS SPME sampling in the central drawer.	40 mg/kg wood
**OBJ4**	Dark chest, painted with still life and year 1853 (Figure 7).	HS SPME on the lid.	<LOD
**OBJ5**	Light chest, painted with birds.	HS SPME inside the chest.	<LOD

In situ HS SPME sampling was carried out on three objects (OBJ3, OBJ4, and OBJ5), but quantitative determination of PCP was possible only for OBJ3. For OBJ4 and OBJ5, the PCP signal was present in the GC–MS chromatograms at the noise level, but a characteristic pattern in the *m/z* range 262–270 was noticeable in both chromatograms. For OBJ3, the PCP signal was evaluated from the HS SPME calibration curves obtained from model wood samples and water solutions. The amount of PCP in the wood was estimated to be 40 mg/kg wood, and the PCP concentration in air was estimated to be 3 ng/m^3^. These results are a rough estimate due to the large differences between the sampling conditions in the museum and in the laboratory: the temperature in the museum depot was lower (approx. 18 °C), the object was coated in paint and varnish, the type of wood was probably neither beech nor spruce, and the weight and volume of the object was much larger compared to the model wood samples. All of these factors could importantly contribute to the distribution of PCP between wood, air, and SPME fiber and consequently affect the results. It has been recently shown that SPME sampling in open areas of museums can lead to very high RSDs [57]. In addition to PCP, lindane and dibutyl phthalate (DBP) were also identified in the HS SPME chromatogram based on NIST spectral library (Figure 8). This confirms that both PCP and lindane were used to treat this object in the past, while DBP presumably originates from plastic packaging, used to protect objects in the depot.

SPME in contact mode was performed in the laboratory on the two largest surfaces of the piece (trim) from OBJ1, but the PCP concentration was below LOD. Since the piece had to be returned to the museum unaffected, no further testing was performed. The smaller piece of OBJ2 was sacrificial, so both HS SPME with the piece enclosed in a headspace vial and solvent extraction with acetone were done, but no PCP signal was detected.

These results were supplemented by the analysis of acetone extracts of cotton wool swabs used as proxies for the acetone extraction of the wood itself. For OBJ3, the determined mass of PCP collected on the swab was 19 µg, while in the other swabs the PCP mass was 1.0–1.6 µg, which can be considered as the limit of detection. The pesticide lindane was also identified in the swab extract of OBJ3, confirming its use on the collection as indicated in the conservation documentation.

**Figure 6:**
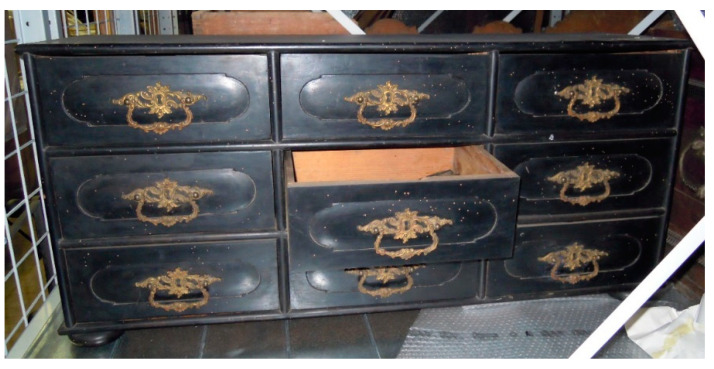
HS SPME sampling was done in the central drawer of OBJ3.

**Figure 8:**
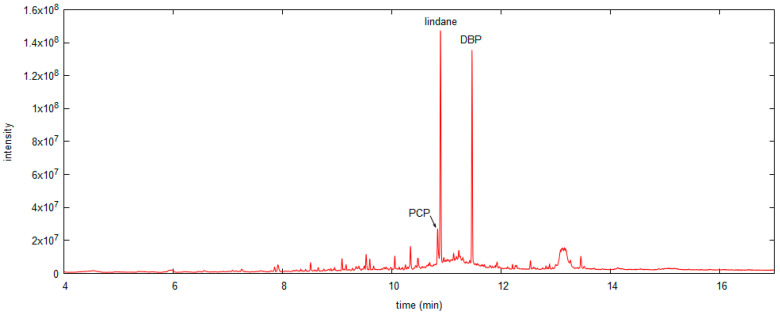
HS SPME chromatogram of OBJ3: PCP, lindane and dibutyl phthalate (DBP) were identified.

From the results for all five wooden objects, OBJ3 clearly stands out, as it is the only one with a PCP amount large enough for quantitative determination. The HS SPME sampling was done in a drawer that had a higher surface-to-volume ratio than the larger chests (OBJ4 and OBJ5). This could contribute to a higher PCP concentration in the air inside a relatively airtight enclosure. Furthermore, as the damage from wood pests is clearly visible on the surface of the object (Figure 6), this object in particular may have undergone additional treatments with PCP in the past that would increase the amount of PCP present in the wood today.

The amount of PCP estimated in OBJ3 (40 mg/kg) fits well within the range of 2–423 mg/kg determined (by a destructive method) in historic wood in a German museum [22]. The recently reported average value of 0.6 mg/kg in Danish furniture waste wood is significantly lower [58], the objects investigated in this study were probably manufactured after the use of PCP was restricted. Traces of PCP were also found in wooden art objects in Belgium (attributed to surface treatment with PCP-impregnated oils), while higher concentrations (possibly due to application by immersion) were detected in wooden printing blocks, but the authors were not able to obtain quantitative data [51].

**Figure 9:**
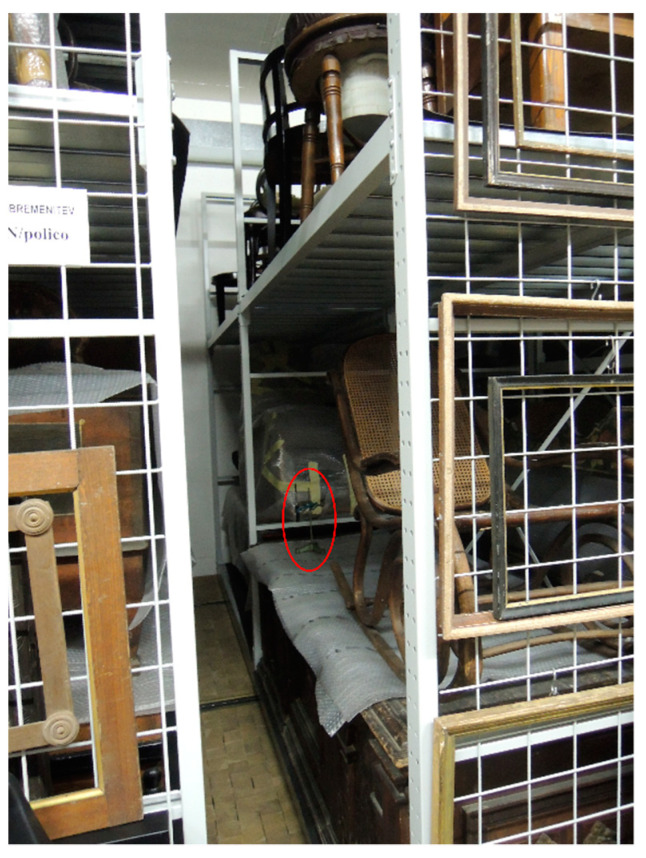
Museum depot air HS SPME sampling location No. 1, the SPME holder circled in red.

Since the presence of PCP in the objects of the Slovenian collection was confirmed, samplings of air in the depot were done to assess the extent of exposure and the impact on the health of the museum employees. HS SPME sampling was done for 4 h at three locations, with the fibers placed at some distance from any of the stored objects (location No. 1 shown in Figure 9).

Small amounts of PCP were detected in locations No. 1 and No. 3. Based on a one-point calibration (4 h HS SPME sampling of a PCP aqueous solution with concentration of 0.1 mg/L, with calculated air concentration of 0.1 ng/m^3^ according to Henry’s law), the air concentrations were estimated to be 0.02 ng/m^3^. Considering the US OSHA concentration limit for PCP in workplace air of 0.5 mg/m^3^ [15], the amount determined in the museum depot is negligible. It is also well below the concentration of 10 ng/m^3^ determined in the indoor air of a museum in Berlin [23]. The results obtained reflect the conditions in the indoor environment at the time of sampling, which may change due to ventilation rate and temperature or with the introduction of a new, highly emitting object, but the PCP concentration in air should currently present no harm through inhalation to the employees entering the depot. Additional caution may be needed when handling treated objects that may contain pesticides in higher amounts, and therefore such objects need to be identified.

## 4. Conclusions

Two non-destructive sampling methods were optimized for the semi-quantitative determination of PCP in wood samples: SPME in headspace or contact mode in combination with GC–MS. Both SPME methods have a linear response in the range of 7.5–75 mg PCP/kg wood and comparable repeatability. The developed methods were used to investigate the presence of PCP in the museum furniture collection depot. Five historical wooden objects were investigated without affecting the objects. Quantitative determination of PCP was possible in one object (40 mg/kg wood), while trace amounts were detected in two objects. Low concentrations of PCP were also detected in the indoor air of the museum furniture depot.

Based on the PCP concentration in the depot air (0.02 ng/m^3^), there is a low health risk for employees according to the limit values of the occupational health and safety authorities, but this should be taken with caution. The concentration of PCP in the depot air can change due to the ventilation rate and temperature and is also dependent on the number of objects. Therefore, monitoring of PCP and other organic compounds in indoor air is recommended.

The SPME sampling methods developed in this study are non-destructive and suitable for semi-quantitative determination of PCP in original wooden museum objects. Further analytical studies would provide more accurate results on real objects, especially with respect to wood mass and type, object surface and surface treatment (e.g., paint, varnish), all of which may influence the distribution of PCP. Nevertheless, the existing methods could be used for a systematic screening of the museum’s wood collections (both in the depot and in the exhibition) to identify possible critical objects and to propose guidelines for a safe handling of treated objects.

## Figures and Tables

**Figure 7:**
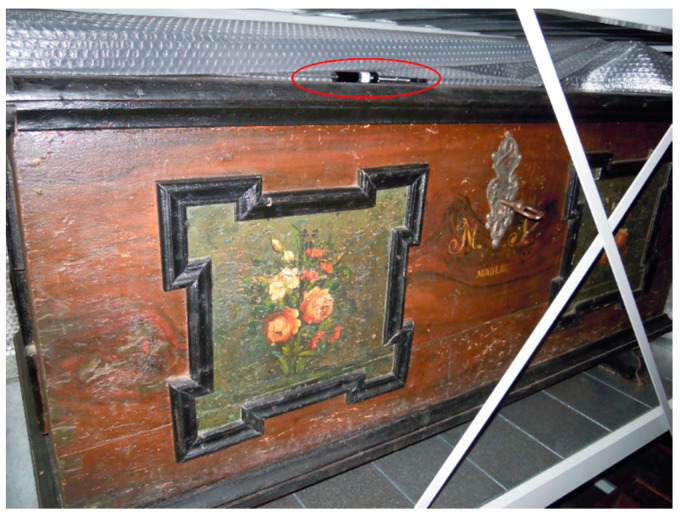
In situ HS SPME sampling of OBJ4, the SPME holder circled in red.

**Table 1:** Comparison of sampling/extraction methods for PCP in wood.

Sampling Mode	Effect on Object/Sample	Sampling Time	* Linearity (γ − PCP Content in Wood (mg/g), Ar − Peak Area, R^2^ − Correlation Coefficient)	** Repeatability (%RSD)	*** LOD (mg PCP/kg Wood)
Headspace SPME	Non-destructive	40 min	Ar = 8.31 × 10^8^ γ − 9.98 × 10^6^ R^2^ = 0.9524	9	20
Contact SPME	Non-destructive	30 min	Ar = 1.41 × 10^9^ γ − 1.15 × 10^7^ R^2^ = 0.8993	16	30
		**Extraction time**	**Extraction recovery (%)**		
Acetone extraction	Destructive	24 h	84	11	10
Acetone/H_2_SO_4_ extraction	Destructive	24 h	66	5	10

* Linearity range 7.5–75 mg/kg; ** Repeatability of SPME in duplicate, solvent extraction in triplicate; *** LOD of solvent extraction estimated from instrumental LOD and recoveries.

## Data Availability

The data generated during the current study are available from the corresponding author on reasonable request.

## References

[1] Unger A., Schniewind A., Unger W. (2001). Conservation of Wood Artifacts: A Handbook.

[2] British Standards Institution (2012). Specification for Managing Environmental Conditions for Cultural Collections.

[3] Nguyen H., Lagarde F., Louarn G., Daniel P. (2017). A New Way to Discriminate Polluted Wood by Vibrational Spectroscopies. Talanta.

[4] Fierascu R.C., Doni M., Fierascu I. (2020). Selected Aspects Regarding the Restoration/Conservation of Traditional Wood and Masonry Building Materials: A Short Overview of the Last Decade Findings. Appl. Sci..

[5] Smithsonian Museum Conservation Institute Furniture Care and Handling. https://www.si.edu/mci/downloads/taking_care/MCIFurnitureCare.pdf.

[6] Gahan C.J. (1920). Furniture Beetles.

[7] Schiopu N., Tiruta-Barna L., Pacheco-Torgal F., Jalali S., Fucic A. (2012). Wood preservatives. Toxicity of Building Materials.

[8] Ferizli A.G., Emekci M. Museum Fumigation. Proceedings of the 2008 Annual International Research Conference on Methyl Bromide Alternatives and Emissions Reductions.

[9] Rushworth I.D., Higgitt C., Smith M., Gibson L.T. (2014). Non-Invasive Multiresidue Screening Methods for the Determination of Pesticides in Heritage Collections. Herit. Sci..

[10] Kearney T. (2001). Chemical Contamination of Repatriated Native Californian NAGPRA Materials: Principles of Risk Assessment for Acute and Chronic Health Effects. Collect. Forum..

[11] Pinniger D.B., Child R.E. Woodworm-a Necessary Case for Treatment? New Techniques for the Detection and Control of Furniture Beetle. Proceedings of the Second International Conference on Urban Pests.

[12] Wörle M., Hubert V., Hildbrand E., Hunger K., Lehmann E., Mayer I., Petrak G., Pracher M., von Arx U., Wülfert S. (2012). Evaluation of Decontamination Methods of Pesticide Contaminated Wooden Objects in Museum Collections: Efficiency of the Treatments and Influence on the Wooden Structure. J. Cult. Herit..

[13] Humar M. (2004). Zaščita Lesa Danes—Jutri (Wood Preservation Today—Tomorrow). Les.

[14] Morrell J.J., Kutz M. (2018). Protection of Wood-Based Materials. Handbook of Environmental Degradation of Materials.

[15] Agency for Toxic Substances and Disease Registry of US Department of Health and Human Services (2001). Toxicological Profile for Pentachlorophenol.

[16] Environmental Protection Agency (2005). Technical Factsheet on: Pentachlorophenol.

[17] Buhr A., Genning C., Salthammer T. (2000). Trace Analysis of Pentachlorophenol (PCP) in Wood and Wood-Based Products—Comparison of Sample Preparation Procedures. Fresenius J. Anal. Chem..

[18] Zhao D. (2014). Determination of Pentachlorophenol Residue in Meat and Fish by Gas Chromatography–Electron Capture Detection and Gas Chromatography–Mass Spectrometry with Accelerated Solvent Extraction. J. Chromatogr. Sci..

[19] Schnelle-Kreis J., Scherb H., Gebefügi I., Kettrup A., Weigelt E. (2000). Pentachlorophenol in Indoor Environments. Correlation of PCP Concentrations in Air and Settled Dust from Floors. Sci. Total Environ..

[20] ARSO (2005). Operativni Program Preprečevanja Onesnaževanja Vodnega Okolja z Nevarnimi Kloriranimi Ogljikovodiki Iz Razpršenih Virov Onesnaževanja.

[21] Inoue K., Yoshida S., Nakayama S., Ito R., Okanouchi N., Nakazawa H. (2006). Development of Stable Isotope Dilution Quantification Liquid Chromatography–Mass Spectrometry Method for Estimation of Exposure Levels of Bisphenol A, 4-Tert-Octylphenol, 4-Nonylphenol, Tetrabromobisphenol A, and Pentachlorophenol in Indoor Air. Arch. Environ. Contam. Toxicol..

[22] Schieweck A., Delius W., Siwinski N., Vogtenrath W., Genning C., Salthammer T. (2007). Occurrence of Organic and Inorganic Biocides in the Museum Environment. Atmos. Environ..

[23] Deering K., Spiegel E., Quaisser C., Nowak D., Schierl R., Bose-O’Reilly S., Garí M. (2019). Monitoring of Arsenic, Mercury and Organic Pesticides in Particulate Matter, Ambient Air and Settled Dust in Natural History Collections Taking the Example of the Museum Für Naturkunde, Berlin. Environ. Monit. Assess..

[24] Wang X., Chen R., Luan T., Lin L., Zou S., Yang Q. (2012). Full Automatic Determination of Chlorophenols in Water Using Solid-Phase Microextraction/on-Fiber Derivatization and Gas Chromatography-Mass Spectrometry: Sample Preparation. J. Sep. Sci..

[25] Amendola L., Cortese M., Vinatoru D., Sposato S., Insogna S. (2017). Innovative Analytical Method for the Determination of Underivatized Tributyltin and Pentachlorophenol in Seawater by Gas Chromatography-Triple Quadrupole Mass Spectrometry. Anal. Chim. Acta.

[26] De Morais P., Stoichev T., Basto M.C.P., Carvalho P.N., Vasconcelos M.T.S.D. (2011). A Headspace SPME-GC-ECD Method Suitable for Determination of Chlorophenols in Water Samples. Anal. Bioanal. Chem..

[27] Liu Y., Wen B., Shan X. (2006). Determination of Pentachlorophenol in Wastewater Irrigated Soils and Incubated Earthworms. Talanta.

[28] Hong H.C., Zhou H.Y., Luan T.G., Lan C.Y. (2005). Residue of Pentachlorophenol in Freshwater Sediments and Human Breast Milk Collected from the Pearl River Delta, China. Environ. Int..

[29] Reigner B.G., Rigod J.F., Tozer T.N. (1990). Simultaneous Assay of Pentachlorophenol and Its Metabolite, Tetrachlorohydroquinone, by Gas Chromatography without Derivatization. J. Chromatogr. B Biomed. Sci. Appl..

[30] Mardones C., Palma J., Sepúlveda C., Berg A., von Baer D. (2003). Determination of Tribromophenol and Pentachlorophenol and Its Metabolite Pentachloroanisole InAsparagus Officinalis by Gas Chromatography/Mass Spectrometry. J. Sep. Sci..

[31] Zhou Y., Jiang Q., Peng Q., Xuan D., Qu W. (2007). Development of a Solid Phase Microextraction-Gas Chromatography–Mass Spectrometry Method for the Determination of Pentachlorophenol in Human Plasma Using Experimental Design. Chemosphere.

[32] Cline R.E., Hill R.H., Phillips D.L., Needham L.L. (1989). Pentachlorophenol Measurements in Body Fluids of People in Log Homes and Workplaces. Arch. Environ. Contam. Toxicol..

[33] Wagner S.L., Durand L.R., Inman R.D., Kiigemagi U., Deinzer M.L. (1991). Residues of Pentachlorophenol and Other Chlorinated Contaminants in Human Tissues: Analysis by Electron Capture Gas Chromatography and Electron Capture Negative Ion Mass Spectrometry. Arch. Environ. Contam. Toxicol..

[34] Zheng W., Wang X., Yu H., Tao X., Zhou Y., Qu W. (2011). Global Trends and Diversity in Pentachlorophenol Levels in the Environment and in Humans: A Meta-Analysis. Environ. Sci. Technol..

[35] Odegaard N., Sadongei A., Boyer L.V. (2005). Old Poisons, New Problems: A Museum Resource for Managing Contaminated Cultural Materials.

[36] Tello H. (2006). Investigations on Super Fluid Extraction (SFE) with Carbon Dioxide on Ethnological Materials and Objects Contaminated with Pesticides. Ph.D. Thesis.

[37] Sirois J.P., Sansoucy G. (2001). Analysis of Museum Objects for Hazardous Pesticide Residues: A Guide to Techniques. Collect. Forum..

[38] Gremaud E., Turesky R.J. (1997). Rapid Analytical Methods To Measure Pentachlorophenol in Wood. J. Agric. Food Chem..

[39] Portoni F., Grau-Bové J., Strlič M. (2019). Application of a Non-Invasive, Non-Destructive Technique to Quantify Naphthalene Emission Rates from Museum Objects. Herit. Sci..

[40] Kearney M., Parkin I., Townsend J.H., Hidalgo M., Curran K. (2018). Characterisation of VOCs Surrounding Naum Gabo’s Construction in Space ‘Two Cones’, (Tate) by in Situ SPME GC-MS Monitoring. Stud. Conserv..

[41] Ormsby M., Johnson J.S., Heald S., Chang L., Bosworth J. (2006). Investigation of Solid Phase Microextraction Sampling for Organic Pesticide Residues on Museum Collections. Collect. Forum..

[42] Alvarez-Martin A., George J., Kaplan E., Osmond L., Bright L., Newsome G.A., Kaczkowski R., Vanmeert F., Kavich G., Heald S. (2020). Identifying VOCs in Exhibition Cases and Efflorescence on Museum Objects Exhibited at Smithsonian’s National Museum of the American Indian-New York. Herit. Sci..

[43] Alvarez-Martin A., McHugh K., Martin C., Kavich G., Kaczkowski R. (2020). Understanding Air-Tight Case Environments at the National Museum of the American Indian (Smithsonian Institution) by SPME-GC-MS Analysis. J. Cult. Herit..

[44] Goewie C.E., Berkhof R.J., Maris F.A., Treskes M., Brinkman U.T. (1986). Determination of Pentachlorophenol in Wood Samples Using Liquid Chromatography With UV Absorbance, Amperometric and Electron-Capture Detection. Int. J. Environ. Anal. Chem..

[45] Pohlandt K., Bockelmann C., Marutzky R. (1995). Concentrations of Pentachlorophenol and Lindane in Various Assortments of Wood. Chemosphere.

[46] Bartelt G., Buge H.G., Görner W., Win T. (1998). Determination of Chlorine and Pentachlorophenol in Wood. Fresenius J. Anal. Chem..

[47] Becker R., Buge H.-G., Win T. (2002). Determination of Pentachlorophenol (PCP) in Waste Wood—Method Comparison by a Collaborative Trial. Chemosphere.

[48] Besner A., Gilbert R., Tetreault P., Lepine L., Archambault J.-F. (1995). Determination of Pentachlorophenol and Its Hydrocarbon Solvent in Wood, Soil, and Water by Gas Chromatography and FT-LR Spectroscopy in a Single-Sample Treatment. Anal. Chem..

[49] European Committee for Standardization (2003). Durability of Wood and Wood-Based Products—Quantitative Determination of Pentachlorophenol in Wood—Gas Chromatographic Method.

[50] Koyano S., Ueno D., Yamamoto T., Kajiwara N. (2019). Concentrations of POPs Based Wood Preservatives in Waste Timber from Demolished Buildings and Its Recycled Products in Japan. Waste Manag..

[51] Covaci A., Kawaki P., Indekeu C., Schepens P., Neels H. (2006). Highly Chlorinated Toxic Contaminants in Pesticide-Treated Wooden Art Objects. Arch. Environ. Occup. Health.

[52] Mauruschat D., Schumann A., Meinlschmidt P., Gunschera J., Salthammer T. (2014). Application of Gas Chromatography—Field Asymmetric Ion Mobility Spectrometry (GC-FAIMS) for the Detection of Organic Preservatives in Wood. Int. J. Ion Mobil. Spec..

[53] Mayer I., Hunger K. Destructive and Non-Destructive Methods for the Evaluation of Chlorinated Pesticides Concentration and Emissions from Wooden Art Objects. Proceedings of the International Conference on Wooden Cultural Heritage: Evaluation of Deterioration and Management of Change.

[54] PubChem Pentachlorophenol. https://pubchem.ncbi.nlm.nih.gov/compound/992.

[55] Sander R. (2015). Compilation of Henry’s Law Constants (Version 4.0) for Water as Solvent. Atmos. Chem. Phys..

[56] Mauruschat D., Plinke B., Aderhold J., Gunschera J., Meinlschmidt P., Salthammer T. (2016). Application of Near-Infrared Spectroscopy for the Fast Detection and Sorting of Wood–Plastic Composites and Waste Wood Treated with Wood Preservatives. Wood Sci. Technol..

[57] Kearney M., Townsend J.H., Parkin I.P., Hidalgo M., Curran K. (2020). Factors Affecting the Practicality of Solid-Phase Microextraction VOC Analysis of Artworks Featuring Polymeric Materials in Open Environments. Microchem. J..

[58] Faraca G., Boldrin A., Astrup T. (2019). Resource Quality of Wood Waste: The Importance of Physical and Chemical Impurities in Wood Waste for Recycling. Waste Manag..

